# Left and right ventricular strain–volume/area loops: an evaluation of intra-observer, inter-observer, and test–retest reliability

**DOI:** 10.1093/ehjimp/qyag069

**Published:** 2026-04-16

**Authors:** Stijn C M Donker, Joseph D Maxwell, Hikmat J Haibe, Elke C C Verhoeven, Thijs P Kerstens, Benjamin J R Buckley, Dick H J Thijssen, David Oxborough

**Affiliations:** Research Institute for Sport and Exercise Sciences, Liverpool John Moores University, Tom Reilly Building, Byrom St, Liverpool L3 3AF, UK; Department of Medical BioSciences, Radboud University Medical Center, Geert Grooteplein Zuid 10, Nijmegen 6525 GA, The Netherlands; Research Institute for Sport and Exercise Sciences, Liverpool John Moores University, Tom Reilly Building, Byrom St, Liverpool L3 3AF, UK; Department of Medical BioSciences, Radboud University Medical Center, Geert Grooteplein Zuid 10, Nijmegen 6525 GA, The Netherlands; Department of Medical BioSciences, Radboud University Medical Center, Geert Grooteplein Zuid 10, Nijmegen 6525 GA, The Netherlands; Department of Internal Medicine, Deventer Ziekenhuis, Nico Bolkensteinlaan 75, Deventer 7416 SE, The Netherlands; Research Institute for Sport and Exercise Sciences, Liverpool John Moores University, Tom Reilly Building, Byrom St, Liverpool L3 3AF, UK; Research Institute for Sport and Exercise Sciences, Liverpool John Moores University, Tom Reilly Building, Byrom St, Liverpool L3 3AF, UK; Department of Medical BioSciences, Radboud University Medical Center, Geert Grooteplein Zuid 10, Nijmegen 6525 GA, The Netherlands; Research Institute for Sport and Exercise Sciences, Liverpool John Moores University, Tom Reilly Building, Byrom St, Liverpool L3 3AF, UK

**Keywords:** echocardiography, deformation imaging, reliability, serial evaluation, strain-volume, strain-area

## Abstract

**Introduction:**

Recent studies highlight that left ventricular (LV) and right ventricular (RV) strain–volume/area interactions, particularly systolic slope and coupling parameters, carry clinical and physiological relevance. This study evaluated the intra-observer, inter-observer, and test–retest reliability of echocardiographic LV and RV strain–volume/area loops.

**Methods and results:**

Twenty-nine healthy adults underwent two transthoracic echocardiograms 2 h apart after standardized preparation. One observer analysed the first scan twice (intra-observer reliability) and the second scan once (test–retest reliability). A second observer analysed the first scan once (inter-observer reliability). Observers were blinded and analysed data independently. Reliability was assessed for systolic (systolic slope [SS], peak strain [PS]) and coupling parameters (early [EarlyU] and late diastolic uncoupling [LateU]), using intra-class correlation coefficients (ICCs) and Bland–Altman analyses. ICCs were generally higher for LV strain–volume than for RV strain–area loops. For LV, intra-/inter-observer and test–retest reliability was good-to-excellent for SS (ICCs: 0.84–0.92), moderate-to-good for PS and EarlyU (ICCs: 0.64–0.85 and 0.60–0.87, respectively), and poor-to-good for LateU (ICCs: 0.48–0.78). For RV, reliability was good for SS (ICCs: 0.78–0.89), poor-to-moderate for PS (ICCs: 0.19–0.59), moderate for EarlyU and LateU (ICCs: 0.53–0.68, and 0.60–0.73, respectively). Systematic bias was minimal.

**Conclusion:**

Reliability was superior for LV-based parameters compared to RV. Both the LV and RV loops showed moderate-to-excellent reliability for SS and EarlyU, whilst reliability for PS and LateU varied from poor-to-good. These findings provide a methodological basis for future studies applying strain–volume and strain–area loops.

## Graphical Abstract

**Graphical Abstract:**
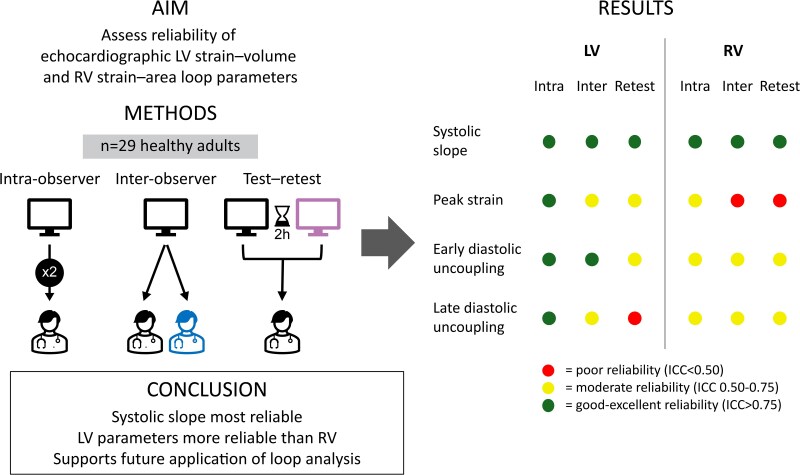

## Introduction

Echocardiography provides comprehensive structural and functional characterization of the heart and is widely applied in cardiology. Contemporary echocardiographic evaluation typically relies on static measures such as ejection fraction and peak strain.^1,2^ However, cardiac function depends on the dynamic interaction between myocardial contraction and loading, which may challenge the interpretation of cardiac performance when assessed by static echocardiographic measures.^1,3,4^ To address this, the strain–volume relationship (SVR) was introduced. The SVR is derived from speckle-tracking echocardiography and captures the interplay between myocardial deformation and ventricular volume throughout the cardiac cycle, thereby providing a dynamic, load-dependent perspective on cardiac mechanics.^3,5,6^

In recent years, several studies have explored the potential of the left ventricular (LV) strain–volume loop (SVL) and right ventricular (RV) strain–area loop (SAL) to assess the SVR in health and disease.^7^ These studies suggest that SVL and SAL analyses are responsive to (patho)physiological stimuli and can distinguish healthy individuals from patient groups (e.g. heart failure, pulmonary hypertension, aortic stenosis).^8–10^ Moreover, the SVL and SAL may be associated with clinical outcomes, independent of conventional static echocardiographic measures.^10–12^ To date, relatively little is known about the reliability of the SVL and SAL analyses. Previous exploratory work assessed intra-observer reliability, but data regarding the inter-observer and test–retest reliability are lacking.^7^

Therefore, our study aims to evaluate the intra-observer, inter-observer, and test–retest reliability of the SVL and SAL in healthy adults. We hypothesize that all SVL and SAL parameters will demonstrate at least moderate-to-good intra-observer, inter-observer, and test–retest reliability. In addition, we expect that the SVL reliability will be generally higher compared to the SAL, given that previous work showed less variation in LV echocardiographic indices compared to the RV.^13^ Establishing the inherent reliability of the SVL and SAL is important to understand (pre)clinical value and practical use.

## Methods

### Population and study design

This prospective cohort study recruited healthy participants aged 20–57 years. Exclusion criteria were the use of prescription medication, tobacco use, a body mass index (BMI) <18 or >30 kg/m^2^, and any chronic medical condition, including systemic hypertension. Ethical approval was granted by Liverpool John Moores University Ethics Research Committee (reference #25/SPS/008), and written informed consent was obtained from all participants prior to enrolment. Participants were recruited through advertisements at Liverpool John Moores University (Liverpool, UK) and attended the study laboratory on a single occasion.

### Measurements

To minimize variation in cardiovascular function and loading conditions between measurements, and to align procedures with previous studies adopting strain–volume analysis, participants were instructed to (1) avoid exercise and caffeine products on the day of the measurements, (2) abstain from alcohol for at least 24 h and recreational drugs for 3 days before the visit, and (3) refrain from eating for ≥2 h before arrival. Measurements were scheduled after 10:00 a.m. to reduce potential influence of the cortisol awakening response.

Personal characteristics (age, sex, adherence to participant instructions) were collected, followed by measurements of weight (Seca 769, Seca, Birmingham, UK) and height (Seca 213, Seca, Birmingham, UK). Following 10 min of supine rest, blood pressure was measured from the left brachial artery using an automated sphygmomanometer (Dinamap Carescape V100, GE Healthcare, Chicago, IL). Systolic blood pressure (SBP), diastolic blood pressure (DBP), and mean arterial pressure (MAP) were recorded twice at 1-min intervals and averaged. Subsequently, participants underwent two transthoracic echocardiograms (TTEs) spaced 2 h apart, while lying in the left lateral decubitus position. Standard and LV focused apical two-, three-, and four-chamber views, along with an RV-focused four-chamber view, were recorded using a commercially available ultrasound system (Vivid E95, GE Healthcare, Chicago, IL) with a 1.5–4 MHz phased array transducer. Frame rate was maintained at ≥50 Hz and kept constant across TTEs within participants, while ensuring visualization of all ventricular wall segments. Heart rate (HR), LV ejection fraction (LVEF) using Simpsons biplane method, and RV fractional area change (RVFAC) were recorded during both TTEs to quantify baseline cardiac function and to confirm physiological comparability, defined as normal LVEF and RVFAC values and <10% absolute deviation between the two echocardiograms. Based on these assessments, all subjects were retained for analysis. All echocardiographic assessments were performed by two UK-registered sonographers (J.M. and D.O.); each participant was examined twice by the same sonographer.

### Post-processing

**Figure 1:**
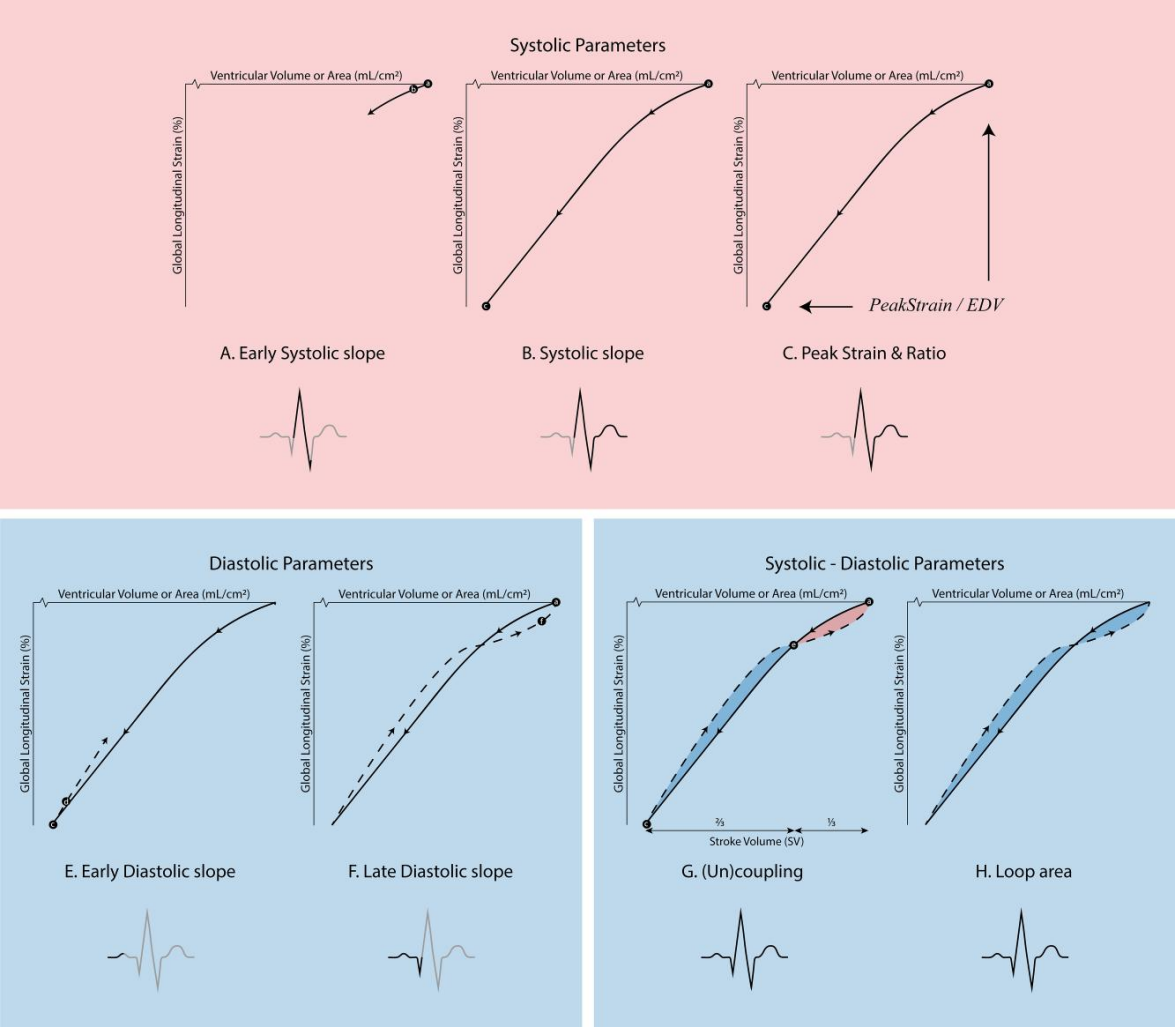
Schematic illustration of loop-derived parameters that describe the relation between strain and volume (LV) or area (RV). Adapted from Kerstens et al. (2024)^7^ under a Creative Commons CC BY license.

Echocardiographic images were analysed offline. To obtain the SVL and SAL parameters, manual strain analysis was performed on images free from artefacts or premature beats. For SVL analysis, apical two-, three-, and four-chamber recordings were used to derive averaged temporal LV global longitudinal strain (GLS) and volumes. For SAL analysis, RV-focused four-chamber recordings were used to derive temporal RV GLS and areas. GLS was calculated as the average longitudinal strain of septal and free-wall segments. Although RV free-wall longitudinal strain is more commonly used in clinical practice than GLS, the interventricular septum contributes substantially to overall right-heart performance, particularly when RV function is impaired.^14–16^ We therefore used GLS to capture total RV deformation. All analyses were conducted across one cardiac cycle using dedicated software (2D Cardiac Performance Analysis v1.4 within ImageArena v4.6; TOMTEC Imaging Systems GmbH, Unterschleißheim, Germany). The resulting strain data were processed by an in-house developed MATLAB script (The MathWorks Inc., version 2019a, MA) to construct the SVL and SAL. In this article, we focus on four parameters that have demonstrated to be of greatest value in previous clinical and physiological studies: (1) systolic slope (SS), (2) peak strain (PS), (3) early diastolic uncoupling (EarlyU), and (4) late diastolic uncoupling (LateU). Less frequently used parameters were presented in Supplementary material S4–S7 (i.e. early systolic slope, early diastolic slope, late diastolic slope, total uncoupling, loop area, and peak strain/end-diastolic volume/area ratio).^8–12^ Together, these parameters characterize different aspects of the strain–volume/area relationship across the cardiac cycle, including systolic function (SS, PS, early systolic slope), diastolic function (early and late diastolic slope) and systolic-diastolic interaction (coupling parameters). *Figure 1* provides an illustration of these parameters, while detailed definitions have previously been described.^7^

Intra-observer reliability was determined by repeated analysis of the first TTE by one observer (SD), inter-observer reliability by independent analysis of the first TTE by a second observer (TK), compared against the first analysis of that scan by SD, and test–retest reliability by analysis of repeated TTE acquisitions by one observer (SD). Both observers had multiple years of experience in SVL/SAL analysis and followed a standard operating procedure that included identification of the end-diastolic and end-systolic frames using the ECG R-wave and the volume/area curve, respectively. Observers were blinded to each other’s results. TTEs were pseudonymized by the sonographer to prevent observers from identifying or matching acquisitions. All strain analyses were performed at least one week after completion of the data-acquisition phase, in a predefined random order generated by study staff independent of the observers using a random number generator. Analyses were conducted on a single workstation under standardized conditions. Together, these steps contributed to a consistent approach and minimized bias.

### Statistics

Baseline data were presented as mean (SD) unless indicated otherwise. Normality of continuous variables was assessed by visual inspection of histograms and Q–Q plots, and Shapiro–Wilk tests. For consistency, absolute values of all SVL and SAL parameters were reported as median and interquartile range.

To assess reliability, intra-class correlation coefficients (ICCs) were computed for each parameter using a two-way mixed-effects model for intra-observer and test–retest reliability, and a two-way random-effects model for inter-observer reliability. Both models used a single-rater type and were defined as ‘absolute agreement’.^17^ A single-rater ICC was chosen to reflect the reliability of single measurements, consistent with clinical practice where the measurements of the SVL/SAL will usually be interpreted individually rather than averaged. The significance of ICCs was derived from the corresponding ANOVA, and 95% confidence intervals were reported. ICC values were interpreted according to the cut-offs proposed by Koo and Li, classifying <0.50 as poor, 0.50–0.75 as moderate, 0.75–0.90 as good, and >0.90 as excellent reliability.^17^ Wilcoxon signed-rank tests were then performed to assess systematic differences in loop parameters within each reliability. Bias was assessed using Bland–Altman plots, including central tendency (mean bias) and the corresponding limits of agreement (LoAs). Finally, post-hoc sensitivity analyses were performed to assess the robustness of findings. We examined whether reliability was influenced by the selection of the cardiac cycle by comparing ICCs of the SVL and SAL parameters from the full cohort with those derived from subsets in which cardiac cycles were matched between repeated analyses for intra- and inter-observer reliability. For the SVL, cases with matched cardiac cycles across at least two apical views (two-, three-, and four-chamber) were retained to maintain sufficient subgroup size. For the SAL, cases with matched cardiac cycles in RV-focused apical four-chamber views were retained.

A two-sided *P*-value <0.05 was considered significant. All participants had complete data. Data processing, visualizations, and statistical analyses were conducted using RStudio version 2024.04.1 (PBC, Boston, MA) with the *psych* and *ggplot2* packages.^18,19^

## Results

### Baseline characteristics

**Figure 2:**
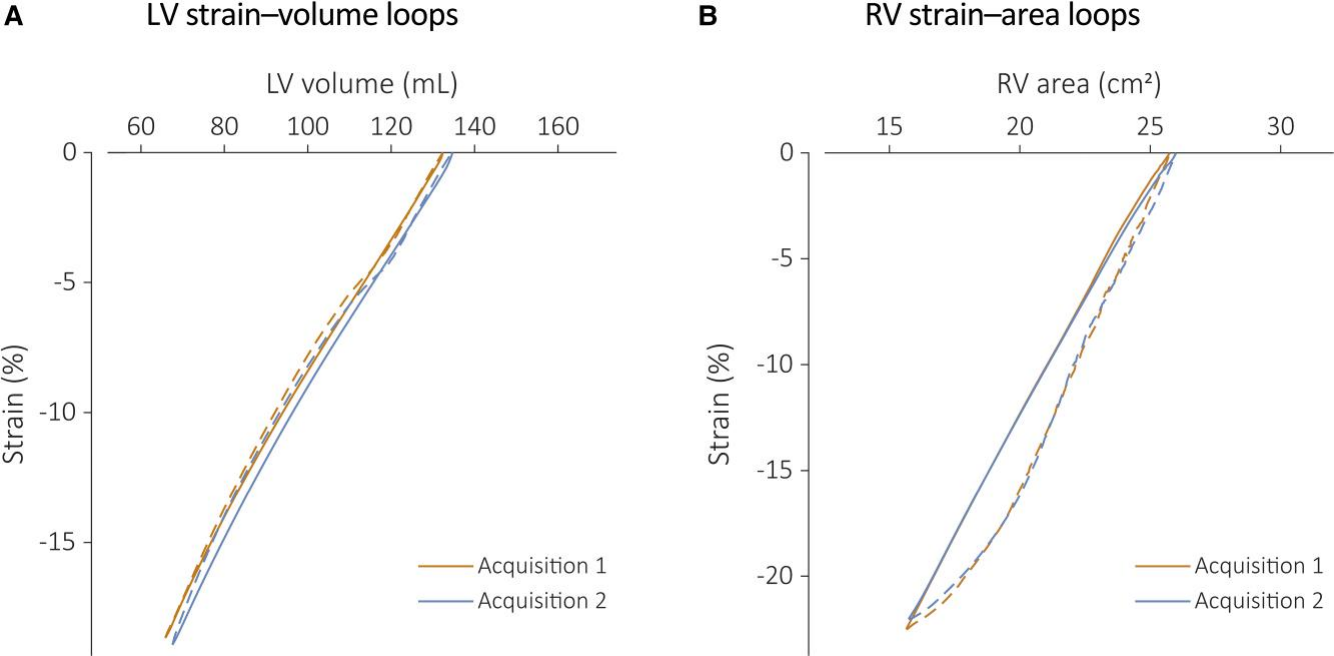
Mean LV strain–volume loops (panel *A*) and RV strain–area loops (panel *B*) obtained from the first and second echocardiographic acquisitions used to assess test–retest reliability (*n* = 29). The continuous line represents the systolic phase of the cardiac cycle; diastole is indicated by the dashed line.

Among 37 screened individuals, 29 were eligible and included in the final analysis. Two were ineligible due to medication use, and six were unable to attend a subsequent study visit. The study population comprised 14 (48%) females and 15 (52%) males with a median age of 27 (25–30) years. Anthropometric and haemodynamic parameters including BMI, BSA, and blood pressure were within normal ranges (*Table 1*). Standard echocardiography confirmed that both LVEF and RVFAC were within reference ranges (59 ± 4% and 44 (43–48)%, respectively; *Table 1*). SVLs and SALs from repeated echocardiographic acquisitions are illustrated in *Figure 2*.

**Table 1:** Baseline characteristics

Variable	Value (*n* = 29)
*Demographics*	
Age (y; median (IQR))	27 (25–30)
Sex (female; *n* (%))	14 (48)
*Anthropometrics*	
Height (m)	1.71 (0.10)
Weight (kg)	70.6 (12.4)
BMI (kg/m^2^)	24.1 (3.1)
BSA (m^2^)	1.82 (0.19)
*Baseline cardiovascular parameters*	
Systolic blood pressure (mmHg)	119 (12)
Diastolic blood pressure (mmHg)	74 (9)
Mean arterial pressure (mmHg)	91 (10)
Heart rate (BPM)	58 (8)
LVEF (%)	59 (4)
RVFAC (%; median (IQR))	44 (43–48)

Baseline characteristics of the study population. Values represent mean (SD) unless indicated otherwise. BPM = beats per minute; BSA = body surface area; IQR = interquartile range.

### SVL reliability and systematic bias

**Figure 3:**
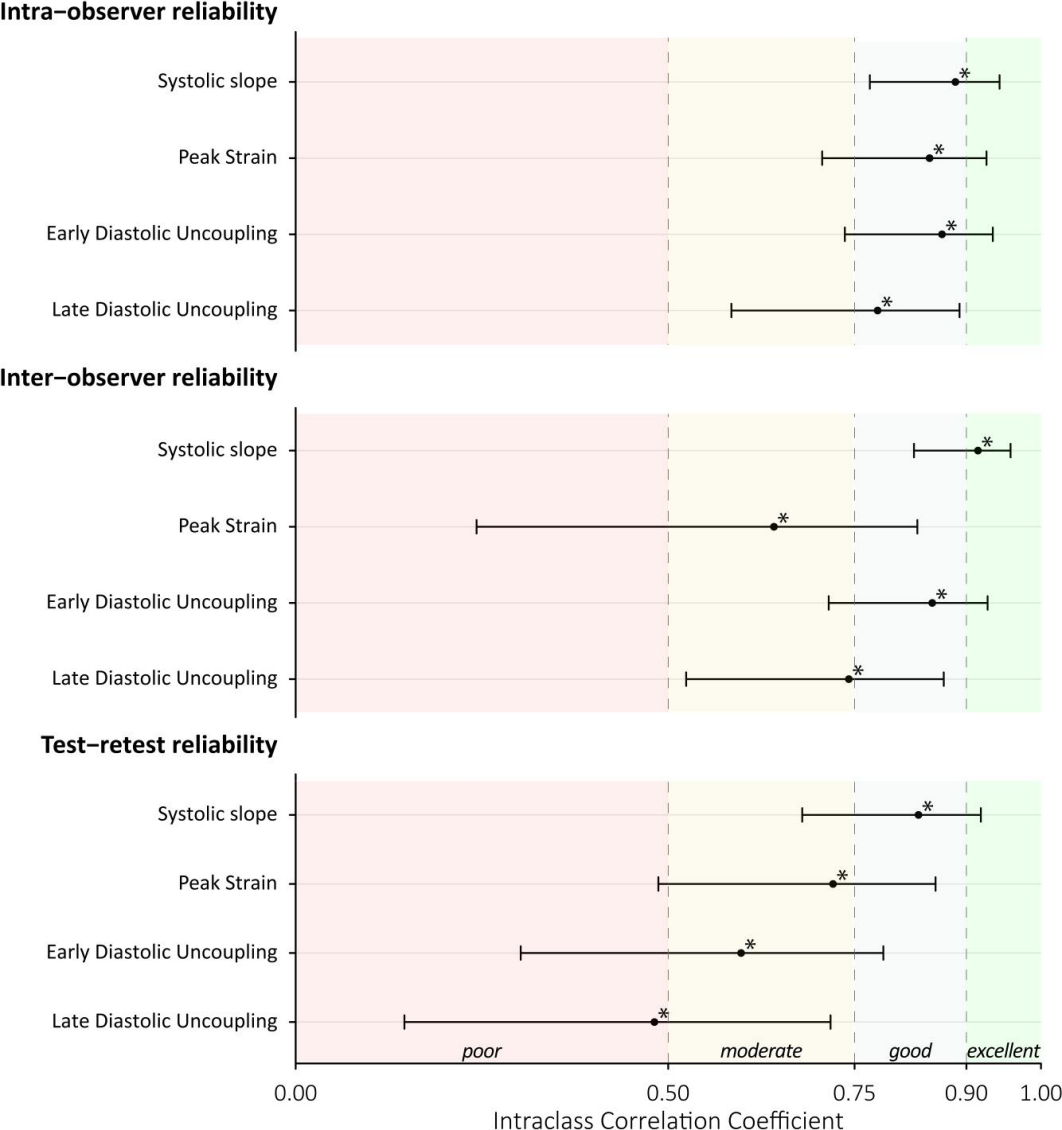
Intra-observer, inter-observer and test–retest reliability of LV strain–volume loop parameters (*n* = 29). Dots represent intra-class correlation coefficients and error bars indicate 95% confidence intervals. Shaded planes illustrate reliability classifications (e.g. ‘poor’, ‘good’). *P < 0.05.

Regarding the reliability of the main SVL parameters, ICCs for the intra-, inter-observer, and test–retest reliability were good-to-excellent for SS (ICCs = 0.89, 0.92, and 0.84; all *P* < 0.001), moderate-to-good for PS (ICCs = 0.85, 0.64 and 0.72; all *P* < 0.001), moderate-to-good for EarlyU (ICCs = 0.87, 0.85 and 0.60; all *P* < 0.001), and poor-to-good for LateU (ICCs = 0.78, 0.74 and 0.48; *P* < 0.001, *P* < 0.001 and *P* = 0.004, respectively) (*Table 2*; *Figure 3*). No significant differences were found between intra-observer assessments (*Table 3*). For inter-observer comparison, a mean systematic bias of −1.19% was found for PS (*P* < 0.001, LoA = −4.60 to 2.21), whilst no significant differences were observed for the other parameters. For test–retest comparison, EarlyU significantly differed between measurements (*P* < 0.05, mean systematic bias −0.34%, LoA = −1.96 to 1.28), with no significant differences for the other parameters. None of the Bland-Altman plots suggested funnel effects (*Table 3*; Supplementary Material  *S1*).

**Table 2:** Reliability of left ventricular strain–volume loop parameters

	Intra-observer (*n* = 29)			Inter-observer (*n* = 29)			Test–retest (*n* = 29)		
Parameter	ICC	95% CI	*P*-value	ICC	95% CI	*P*-value	ICC	95% CI	*P*-value
Systolic slope	0.89*	0.77–0.94	<0.001	0.92*	0.83–0.96	<0.001	0.84*	0.68–0.92	<0.001
Peak strain	0.85*	0.71–0.93	<0.001	0.64*	0.24–0.83	<0.001	0.72*	0.49–0.86	<0.001
Early diastolic uncoupling	0.87*	0.74–0.94	<0.001	0.85*	0.72–0.93	<0.001	0.60*	0.30–0.79	<0.001
Late diastolic uncoupling	0.78*	0.58–0.89	<0.001	0.74*	0.52–0.87	<0.001	0.48*	0.15–0.72	0.004

Intra-, inter-observer, and test–retest reliability for the left ventricular strain–volume loop parameters. CI = confidence interval. *P < 0.05.

**Table 3:** Absolute values of left ventricular strain–volume loop parameters across analyses

	Baseline (*n* = 29)		Intra-observer (*n* = 29)		Inter-observer (*n* = 29)		Test–retest (*n* = 29)	
Parameter	Outcome	IQR	Outcome	IQR	Outcome	IQR	Outcome	IQR
Systolic slope (%/mL)	0.27	0.23–0.33	0.28	0.24–0.32	0.29	0.22–0.34	0.28	0.23–0.35
Peak Strain (%)	−18.43	−20.72 to −17.12	−18.95	−19.80 to −17.33	−19.76*	−21.01 to −18.87	−18.04	−20.23 to −17.49
Early diastolic uncoupling (%)	−0.39	−0.92 to 0.26	−0.57	−1.05 to 0.37	−0.26	−0.90 to 0.12	−0.82*	−1.29 to 0.16
Late diastolic uncoupling (%)	−0.01	−0.57 to 0.51	−0.07	−0.55 to 0.45	−0.09	−0.51 to 0.48	−0.33	−0.63 to 0.54

Absolute outcomes of left ventricular strain–volume loop parameters. Values represent median and interquartile range. Wilcoxon signed-rank tests evaluated differences relative to the baseline analysis (i.e. first assessment of the first TTE by observer 1). Intra-observer: second analysis of the first TTE by observer 1; inter-observer: independent analysis of the first TTE by observer 2; test–retest: analysis of second TTE by observer 1.

IQR = interquartile range; TTE = transthoracic echocardiogram. *P < 0.05.

### SAL reliability and systematic bias

**Figure 4:**
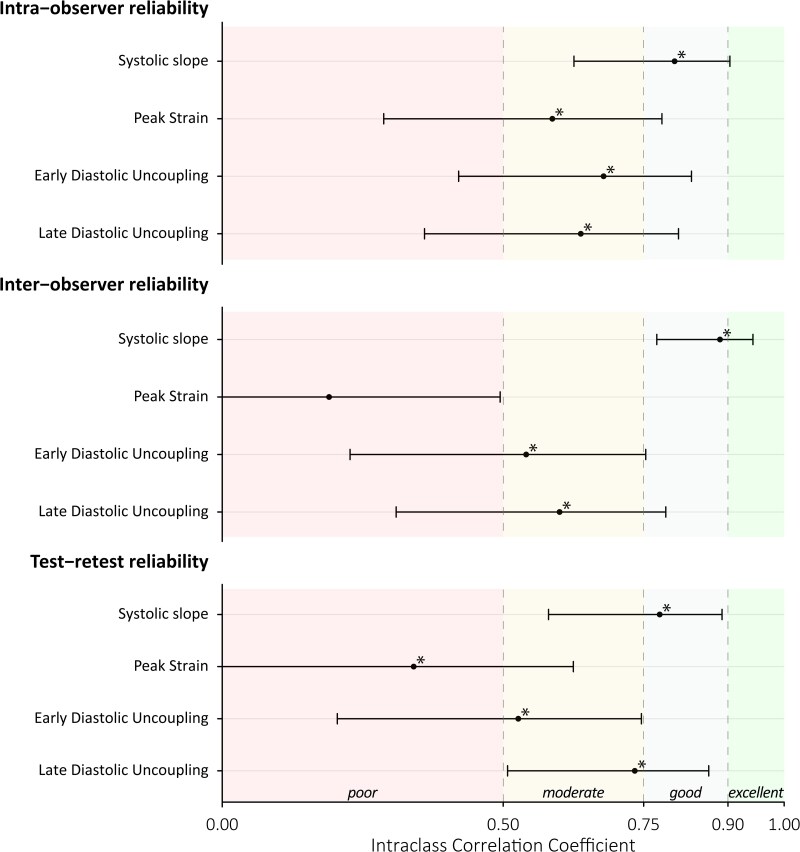
Intra-observer, inter-observer, and test–retest reliability of RV strain–area loop parameters (*n* = 29). Dots represent intra-class correlation coefficients and error bars represent 95% confidence intervals. The delineated coloured planes indicate reliability classifications (e.g. ‘poor’, ‘good’). *P < 0.05.

Regarding the reliability of SAL, ICCs for the intra-, inter-observer, and test–retest reliability were good for SS (ICCs = 0.81, 0.89, and 0.78; all *P* < 0.001), poor-to-moderate for PS (ICCs = 0.59, 0.19, and 0.34; *P* < 0.001, *P* = 0.125, and *P* < 0.001, respectively), moderate for EarlyU (ICCs = 0.68, 0.54, and 0.53; all *P* ≤ 0.001), and moderate for LateU (ICCs = 0.64, 0.60, and 0.73; all *P* < 0.001) (*Table 4*, *Figure 4*). No systematic bias was found for intra-observer and test–retest comparisons of the SAL (*Table 5*). For inter-observer comparisons, only PS differed significantly (*P* = 0.029, mean systematic bias −2.07%, LoA = −10.93 to 6.78) between measurements. Inspection of the Bland–Altman plots suggested no funnel effects (*Table 5*, Supplementary Material S2).

**Table 4:** Reliability of right ventricular strain–area loop parameters

	Intra-observer (*n* = 29)			Inter-observer (*n* = 29)			Test–retest (*n* = 29)		
Parameter	ICC	95% CI	*P*-value	ICC	95% CI	*P*-value	ICC	95% CI	*P*-value
Systolic slope	0.81*	0.63–0.90	<0.001	0.89*	0.77–0.94	<0.001	0.78*	0.58–0.89	<0.001
Peak strain	0.59*	0.29–0.78	<0.001	0.19	−0.13 to 0.49	0.125	0.34*	−0.02 to 0.62	0.033
Early diastolic uncoupling	0.68*	0.42–0.84	<0.001	0.54*	0.23–0.75	0.001	0.53*	0.20–0.75	0.001
Late diastolic uncoupling	0.64*	0.36–0.81	<0.001	0.60*	0.31–0.79	<0.001	0.73*	0.51–0.87	<0.001

Intra-, inter-observer, and test–retest reliability for the right ventricular strain–area loop parameters. CI = confidence interval. *P < 0.05.

**Table 5:** Absolute values of right ventricular strain–area loop parameters across analyses

	*Baseline (n* = *29)*		*Intra-observer (n* = *29)*		*Inter-observer (n* = *29)*		*Test–retest (n* = *29)*	
Parameter	Outcome	IQR	Outcome	IQR	Outcome	IQR	Outcome	IQR
Systolic slope (%/cm^2^)	2.42	1.90–2.77	2.21	1.80–2.62	2.42	2.03–2.66	2.18	1.87–2.59
Peak Strain (%)	−22.54	−23.76 to −21.58	−22.27	−24.57 to −19.61	−24.64*	−26.03 to −21.60	−22.05	−23.94 to −20.85
Early diastolic uncoupling (%)	2.78	1.98–3.22	2.93	2.24–3.28	2.83	1.82–3.73	2.63	1.48–3.94
Late diastolic uncoupling (%)	1.29	0.62–2.44	1.36	0.40–2.34	0.90	0.49–1.85	1.35	0.57–1.93

Absolute outcomes of right ventricular strain–area loop parameters. Values represent median and interquartile range. Wilcoxon signed-rank tests evaluated differences relative to the baseline analysis (i.e. first assessment of the first TTE by observer 1). Intra-observer: second analysis of the first TTE by observer 1; inter-observer: independent analysis of the first TTE by observer 2; test–retest: analysis of second TTE by observer 1.

IQR = interquartile range; TTE = transthoracic echocardiogram. *P < 0.05.

### Post-hoc sensitivity analyses

To evaluate the impact of cardiac cycle selection on reliability, we repeated the analysis in cases with matched cardiac cycles for intra- and inter-observer assessments. For the SVL, subsets for intra-observer (*n* = 20) and inter-observer (*n* = 17) reliability resulted in ICCs comparable to the primary analysis (ΔICC <0.10 for most parameters). For the SAL, intra-observer (*n* = 18) reliability showed somewhat higher ICCs for most parameters compared to the primary analysis, while inter-observer (*n* = 14) reliability was slightly lower for some and higher for other parameters. The largest changes occurred in parameters with an initial ICC <0.25. Overall reliability classification (e.g. ‘poor’ or ‘good’) remained largely unchanged, and most parameters showed a ΔICC between 0.10 and 0.20 (see Supplementary Material S3).

Given the poor-to-moderate reliability of RV PS, we explored whether this is affected by using manual vs. automated analysis. We re-analysed RV GLS using TOMTEC RV AutoStrain and compared intra-observer and test–retest ICCs with those obtained from the manual analysis. This yielded moderate reliability (ICC ≈ 0.70).

## Discussion

This study evaluated the intra-observer, inter-observer, and test–retest reliability of parameters derived from echocardiographic LV SVL and RV SAL in healthy individuals. Three main findings emerged from this study. First, for LV SVLs, the systolic slope was highly reliable, whereas peak strain was less consistent. Both early and late diastolic uncoupling were consistent within and between observers, but showed a somewhat lower reliability across repeated scans. Second, similar patterns were observed for RV SALs, with high reliability for systolic slope, whereas peak strain reliability was poor. Early and late diastolic uncoupling showed moderate, though variable, reliability. Third, most parameters for both LV and RV demonstrated the highest reliability for intra-observer comparisons, followed by inter-observer and test–retest reliability. Together, these data provide a foundation for the interpretation and future application of SVL and SAL analyses.

Reliability of LV SVL parameters followed a hierarchical trend, in that intra-observer reliability was the highest, followed by inter-observer and test–retest reliability. This is consistent with observations in conventional echocardiography.^20–22^ In line with our hypothesis, all SVL parameters, except for LateU, demonstrated moderate-to-good reliability for intra-observer, inter-observer, and test–retest analyses. Considering systolic parameters, the reliability of SS was good-to-excellent for intra-, inter-observer, and test–retest analyses, whilst PS showed only moderate agreement between observers and across repeated scans. This seems paradoxical, as parameters that depend on the assessment of two measures (i.e. slope = strain/Δvolume) are usually prone to more measurement error than direct parameters such as PS.^23^ Possibly, overestimating myocardial shortening leads to overestimating volume reduction within the same cardiac cycle. Such correlated errors may fade each other out when expressed as a ratio. Regarding the coupling parameters, EarlyU was more reliable than LateU, although both showed more variability than SS. Taken together, we identify SS as the most reliable SVL parameter. Encouragingly, SVL characteristics that have previously shown prognostic relevance (i.e. SS and measures of systolic–diastolic uncoupling) were among the more reliable parameters in our dataset.^7,10–12^

RV SAL parameters followed a similar hierarchy across intra-, inter-observer and test–retest reliability types, but with greater overall variability than LV SVL. The larger variability in RV SAL may not be surprising, as conventional echocardiographic measures also vary more for the RV compared to LV.^13^ Regarding individual parameters, SS was reliable whereas PS performed poorly, despite the established clinical value of RV peak strain.^14^ This discrepancy may be attributable to technical factors. Specifically, PS was derived from manual strain analysis, whilst clinical practice relies on automation. Indeed, automated re-analysis of RV GLS substantially improved reliability, which is in line with previous work in this field.^24^ Accordingly, technical challenges such as inconsistent manual RV wall-tracing may contribute to the poor reliability of PS. Regarding coupling parameters, reliability of LateU was higher than that of EarlyU. This contradicts LV results where EarlyU performed more reliably, which may be related to underlying differences in chamber geometry and load dependence. Overall, these findings highlight the methodological consistency of RV SS and LateU, while suggesting that the greater variability observed in RV parameters may be partly technical but also reflects distinct ventricular mechanics. Consequently, RV and LV parameters should not be considered interchangeable.

An important practical consequence of the distinct reliability of the various parameters, as well as the differences between LV and RV, is that these should be considered when planning future studies, particularly when calculating the sample size. In this respect, several technical and practical considerations may help enhance the reliability of SVLs/SALs. Complex RV geometry limits accurate volumetric assessment using two-dimensional (2D) echocardiography, whereas recent developments in 3D echocardiography enable high-frame-rate measurement of RV volumes with simultaneous strain. Early studies demonstrate the feasibility of 3D SVLs/SALs,^25,26^ which may help improve measurement reliability of the SAL. In addition, manual strain analysis as adopted in the present study is time-consuming and introduces variation, and current AutoStrain techniques are not primarily designed to offer synchronized frame-by-frame strain and volumetric/area output required for loop generation. As such, further technical development may improve the feasibility of loop analysis.

Beyond these methodological insights, it is worth noting that several loop parameters have shown diagnostic or prognostic relevance in conditions such as heart failure, pulmonary hypertension, and valvular disease.^7^ For instance, systolic slope differed by 0.4%/cm^2^ between patients with pulmonary hypertension who died during 5-year follow-up compared with survivors.^12^ In the present study, absolute variability across analyses, observers, and serial assessments remained <0.24%/cm^2^, aiding the interpretation of prior loop-based outcomes. Building on this perspective, test–retest reliability carries particular relevance as it reflects the intrinsic variability of serial evaluations and provides the context for interpreting changes in loop-derived parameters. This reliability is inherently lower than intra- or inter-observer reliability because it additionally incorporates acquisition-related factors, including probe positioning and short-term physiological fluctuations in heart rate, rhythm, preload and afterload. A sensitivity analysis excluding individuals with a heart rate difference >10% between scans (*n* = 7) showed similar ICCs (data not shown), suggesting that heart rate differences are unlikely to be a major driver of variability in this healthy cohort. Such physiological factors may play a greater role in clinical populations, which should be considered when interpreting serial changes in patient groups.

From a clinical perspective, SVLs/SALs provide a load-dependent assessment of myocardial function, complementing conventional echocardiographic measures that largely rely on static indices which do not account for loading conditions. By reflecting the dynamic interaction between ventricular mechanics and haemodynamics, loop-based analysis may improve echocardiographic interpretation of cardiac function.^7^ A related echocardiographic approach is myocardial work, which integrates myocardial strain with pressure to assess load-dependent cardiac mechanics.^27^ However, continuous pressure information is not always available, and for the RV, myocardial work relies on estimated pressure curves with limited validation, particularly during diastole.^28,29^ As strain–volume/area analysis can be derived from standard echocardiographic recordings, it offers a complementary, broadly applicable alternative. While the current study does not establish clinical utility of loop-derived parameters, the results provide context for interpreting single and serial evaluations and offer a foundation for future empirical research into their potential clinical value.

### Limitations

A key strength lies in providing the first comprehensive assessment of the reliability of LV strain–volume and RV strain–area relationships, and the minimizing of physiological and procedural variation. We also recognize some limitations. First, reliability research inherently requires a balance between isolating measurement error and reflecting clinical reality. Studying a healthy, relatively young and normal-BMI cohort provides a best-case scenario and reduces avoidable variation, but may also limit generalizability to clinical populations where complex echocardiographic procedures may be more challenging. Second, the present study did not assess reliability under altered physiological conditions such as preload, afterload or heart rate, which may affect measurement reliability in patient groups. In addition, the short interval between repeated measurements means that our test–retest findings reflect intrinsic measurement variability rather than day-to-day reproducibility, where physiological fluctuations may contribute to variability. Third, each observer was allowed to independently select images for post-processing. This may have introduced a degree of variation, but also reflects the conditions under which echocardiography is performed in practice. Reassuringly, the sensitivity analysis showed that cardiac cycle selection only mildly affected reliability. Fourth, although image quality may have varied with body composition, all images met predefined quality criteria. Finally, unintended between-subject variation may have occurred since two sonographers performed the measurements. This may have affected ICC values. Within-subject variation was, however, controlled by keeping sonographers the same for within-participant comparison. Furthermore, between-sonographer differences resemble the clinical reality where echocardiograms are often performed by, and shared among, different health care professionals.

## Conclusion

Although reliability of LV-based loop parameters was marginally higher than that of RV-based parameters, both ventricles demonstrated good-to-excellent intra-observer, inter-observer, and test–retest reliability for systolic slope, and moderate-to-good reliability for early diastolic uncoupling. This is especially relevant since previous studies highlight the potential clinical and/or physiological relevance of these outcome measures. Furthermore, reliability of peak strain and late diastolic uncoupling ranged from poor to good. This work provides a comprehensive evaluation of the reliability of echocardiographic strain–volume and strain–area loops. The insights outlined in our study may guide future research, support technical development, and facilitate clinical implementation of strain–volume and strain–area loops.

## Supplementary Material

qyag069_Supplementary_Data

## Data Availability

The data underlying this article will be shared on reasonable request to the corresponding author.

## Lead author biography

Stijn C.M. Donker is a medical doctor (registered in The Netherlands) and dual PhD candidate at Liverpool John Moores University and Radboud University Medical Centre (The Netherlands). His work combines patient care with clinical research studies, with a particular focus on cardiovascular pathophysiology.
