# Supplementary material for: Left and right ventricular strain–volume/area loops: an evaluation of intra-observer, inter-observer, and test–retest reliability
Source: Eur Heart J Imaging Methods Pract. 2026 Apr 16;4(1):qyag069. doi: 10.1093/ehjimp/qyag069 (PMC13155451; doi:10.1093/ehjimp/qyag069)
Supplement: qyag069_Supplementary_Data [file qyag069_supplementary_data.docx]

**Supplementary material**

**Left and right ventricular strain–volume/area loops: an evaluation of intra-observer, inter-observer and test**–**retest reliability**

Stijn C.M. Donker MD, Joseph D. Maxwell PhD, Hikmat J. Haibe, Elke C.C. Verhoeven, Thijs P. Kerstens MD PhD, Benjamin J.R. Buckley PhD, Dick H.J. Thijssen PhD, David Oxborough PhD

**Supplementary Material 1. Bland-Altman plots of strain–volume loop parameters**

**Supplementary Material 2. Bland-Altman plots of strain–area loop parameters**

**Supplementary Material 3. Sensitivity analyses: reliability of SVL and SAL parameters for matched cardiac cycles**

**Supplementary Material 4. Reliability of all left ventricular strain–volume loop parameters**

**Supplementary Material 5. Absolute values of all left ventricular strain–volume loop parameters across analyses**

**Supplementary Material 6. Reliability of all right ventricular strain–area loop parameters**

**Supplementary Material 7. Absolute values of all right ventricular strain–area loop parameters across analyses**

**Supplementary Material 1. Bland-Altman plots of strain–volume loop parameters**

*Intra-observer comparisons (SVL)*:


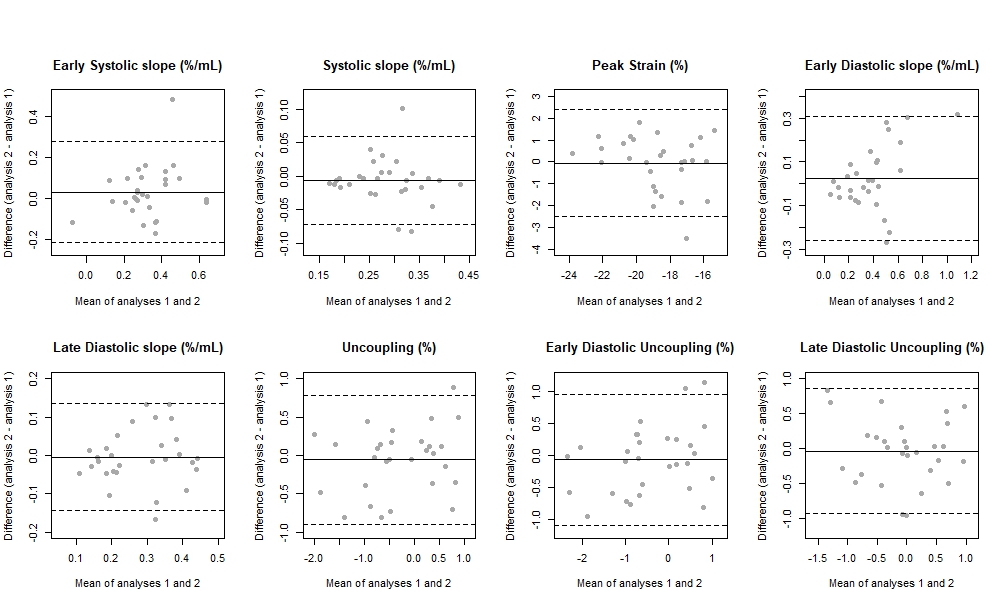


*
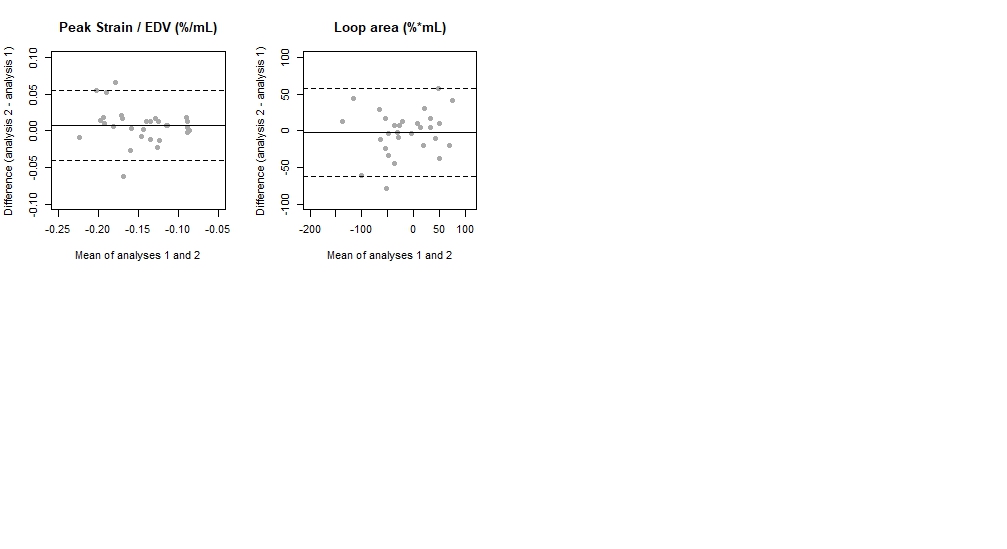
*

*Inter-observer comparisons (SVL)*:

**
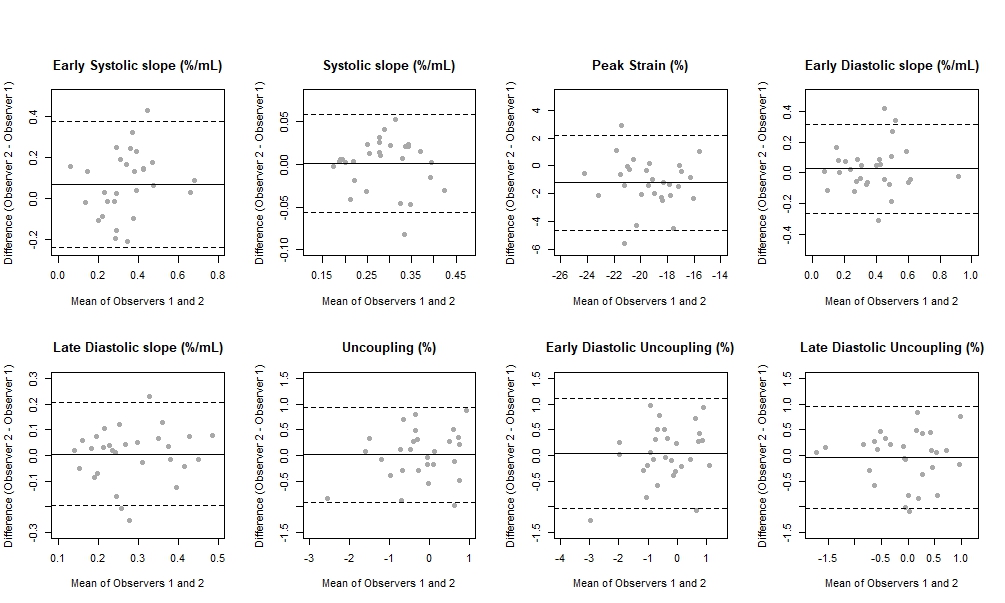
**

**
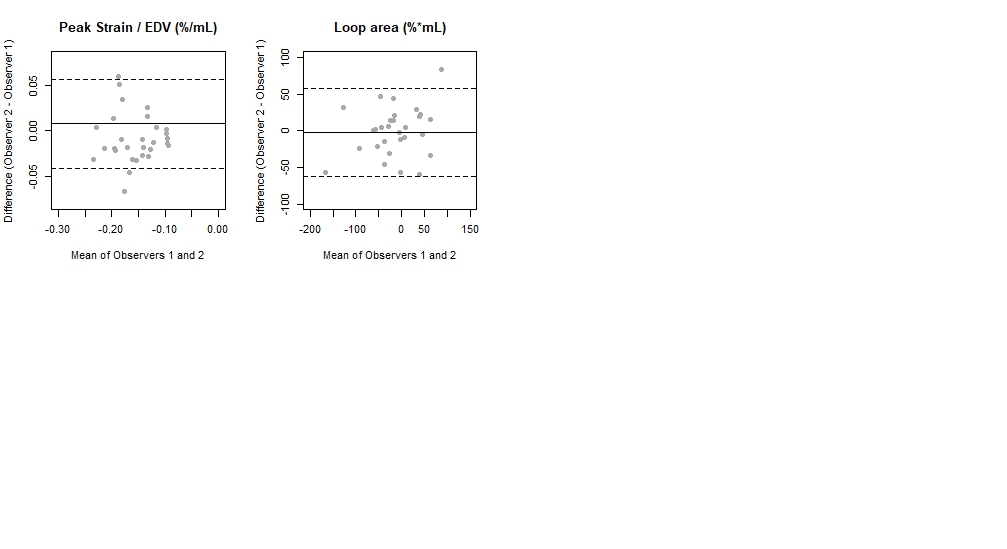
**

*Test–retest comparisons (SVL)*:


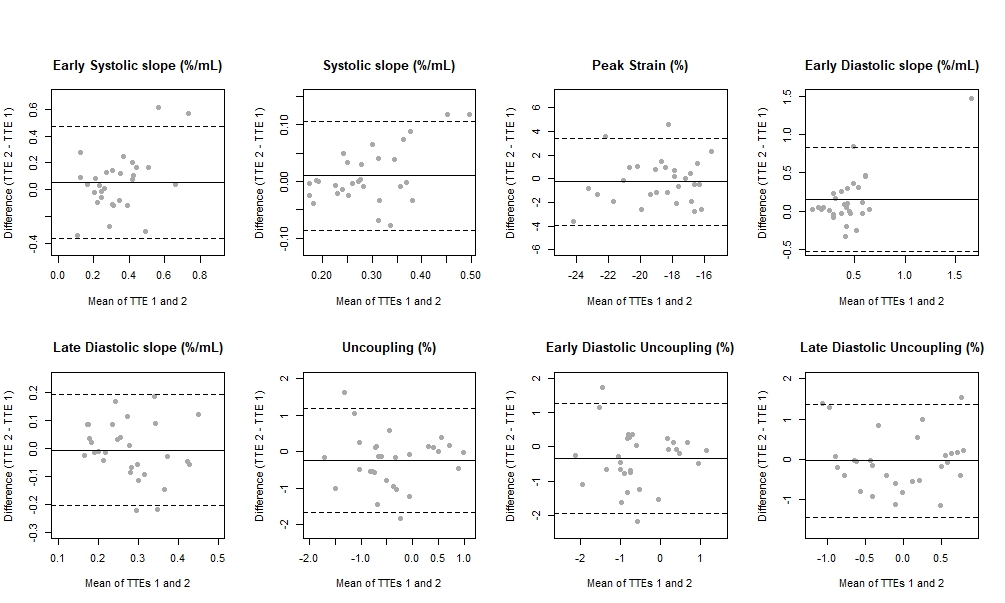


**
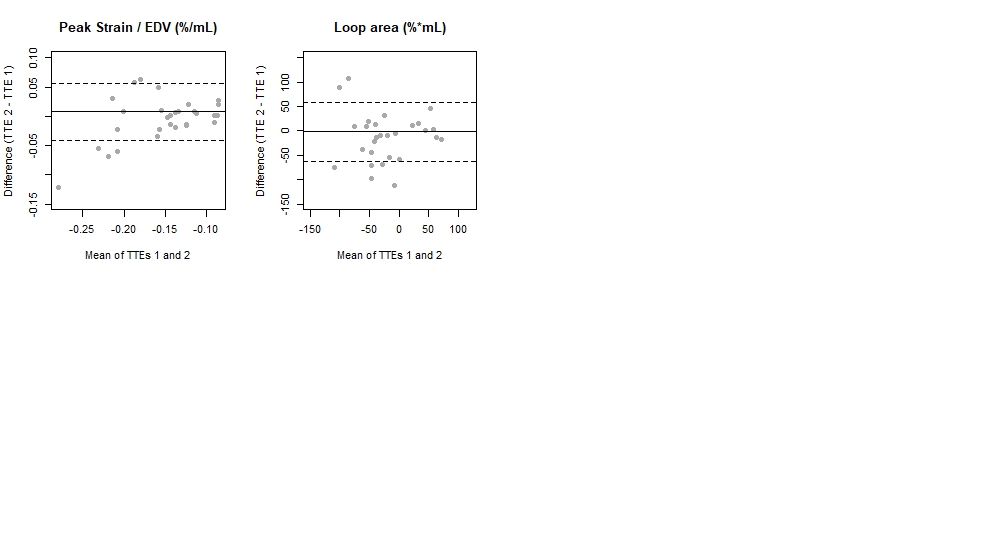
**

**Supplementary Material 2. Bland-Altman plots of strain–area loop parameters**

*Intra-observer comparisons* *(SAL):*


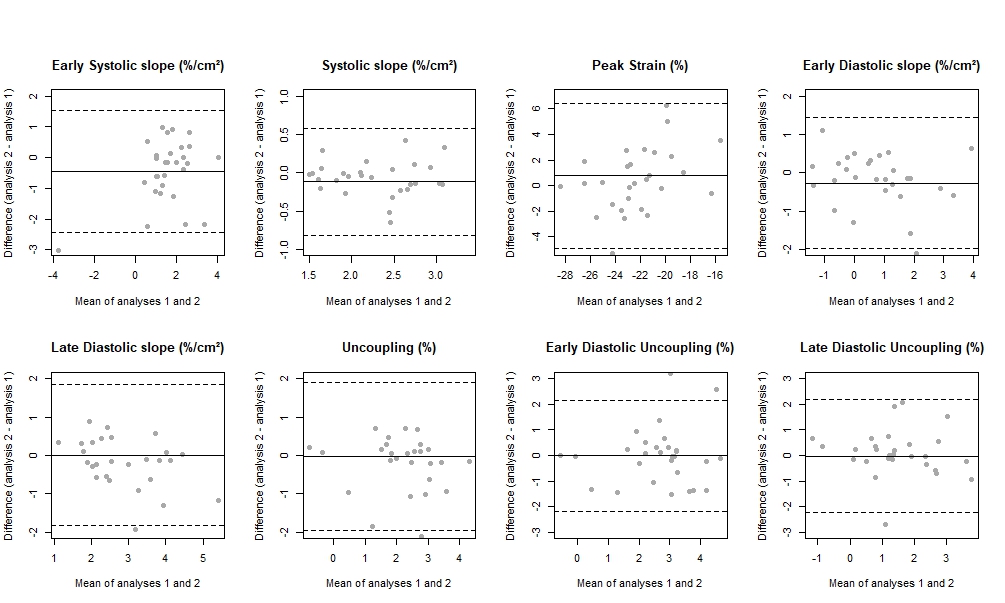


*
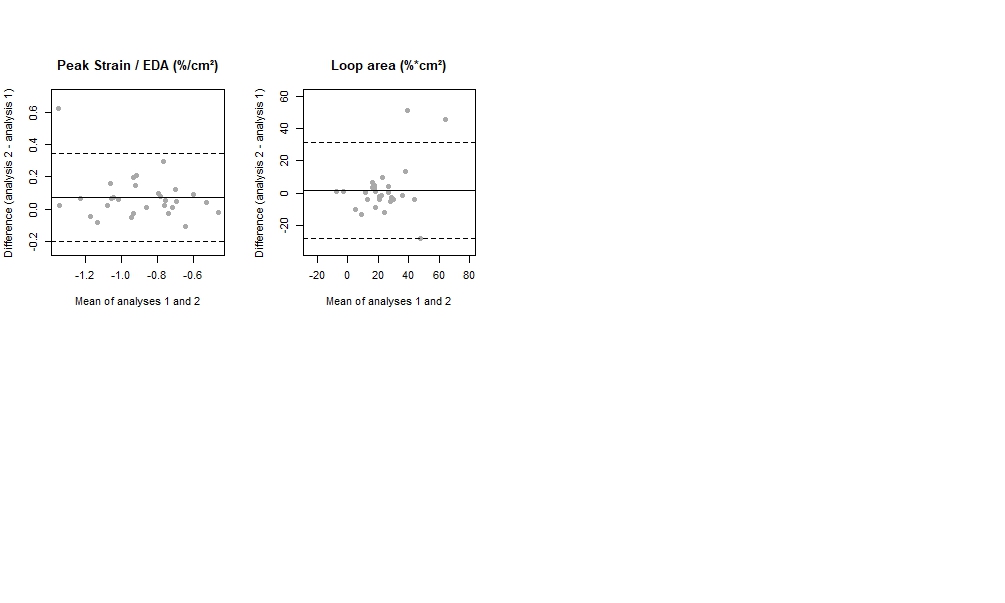
*

*Inter-observer comparisons (SAL):*

*
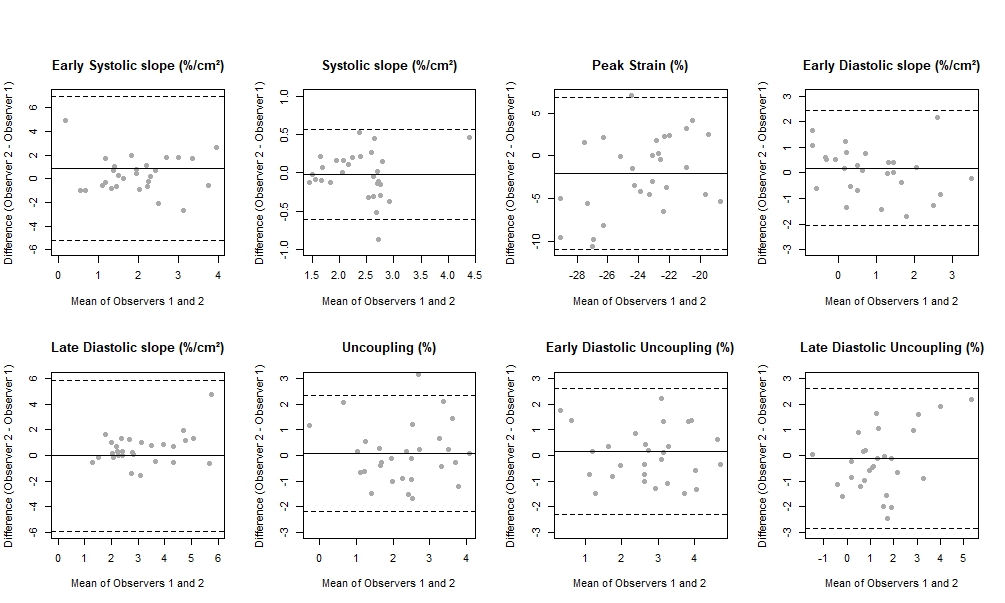
*

*
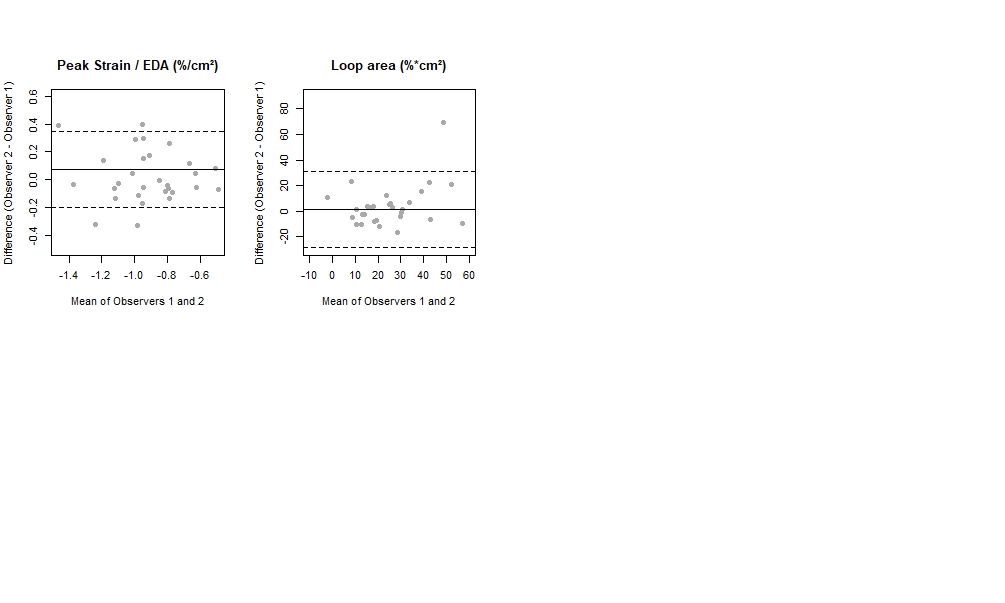
*

*Test–retest comparisons (SAL)*

*
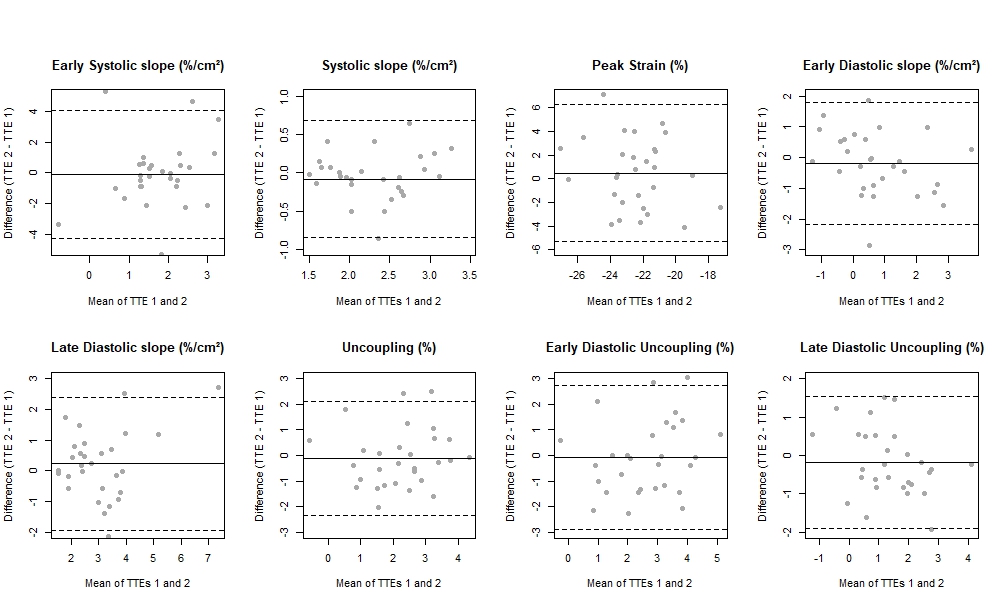
*

**
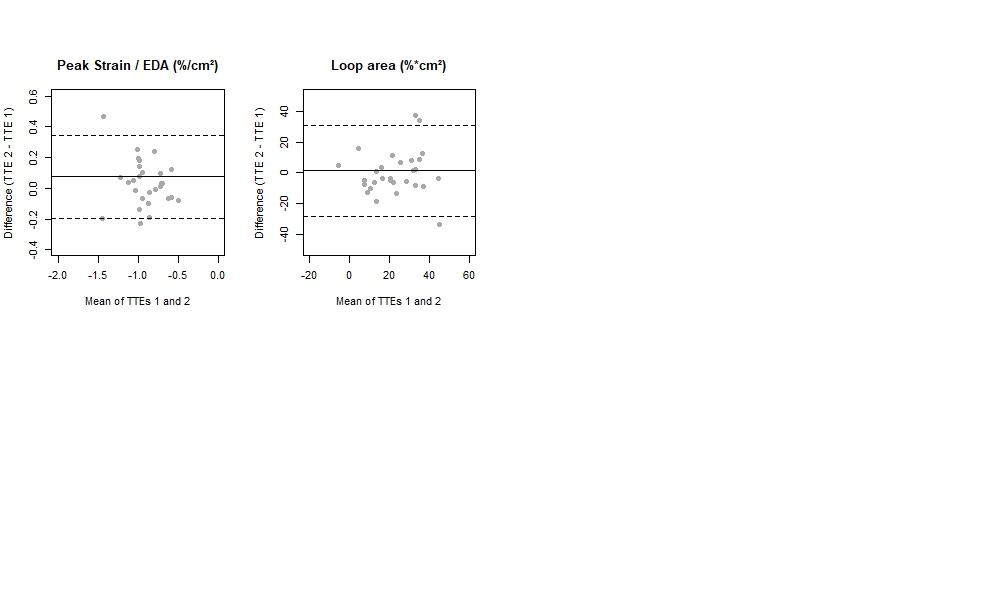
**

**Supplementary Material 3. Sensitivity analyses: reliability of SVL and SAL parameters for matched cardiac cycles**

|  | ***SVL*** | | | | | | ***SAL*** | | | | | | |
| --- | --- | --- | --- | --- | --- | --- | --- | --- | --- | --- | --- | --- | --- |
|  | ***Intra-observer (n=20)*** | | | ***Inter-observer (n=17)*** | | | ***Intra-observer (n=14)*** | | | ***Inter-observer (n=18)*** | | | |
| **Parameter** | **ICC** | **95% CI** | ***p*-value** | **ICC** | **95% CI** | ***p*-value** | **ICC** | **95% CI** | ***p*-value** | **ICC** | **95% CI** | ***p*-value** | |
| Early Systolic slope | 0.83* | 0.63–0.93 | <0.001 | 0.61* | 0.05–0.86 | <0.001 | 0.76* | 0.46–0.90 | <0.001 | 0.23 | -0.19–0.63 | 0.156 | |
| Systolic slope | 0.91* | 0.78–0.96 | <0.001 | 0.96* | 0.90–0.99 | <0.001 | 0.80^*^ | 0.54–0.92 | <0.001 | 0.78* | 0.46–0.92 | <0.001 | |
| Peak Strain | 0.88* | 0.72–0.95 | <0.001 | 0.72* | 0.05–0.91 | <0.001 | 0.72* | 0.39–0.88 | <0.001 | 0.03 | -0.47–0.53 | 0.451 | |
| Early Diastolic slope | 0.77* | 0.51–0.90 | <0.001 | 0.89* | 0.72–0.96 | <0.001 | 0.72* | 0.39–0.88 | <0.001 | 0.53* | 0.03–0.82 | 0.022 | |
| Late Diastolic slope | 0.83* | 0.62–0.93 | <0.001 | 0.70* | 0.28–0.88 | <0.001 | 0.68* | 0.32–0.87 | <0.001 | 0.82* | 0.53–0.94 | <0.001 | |
| Uncoupling | 0.85* | 0.66–0.94 | <0.001 | 0.91* | 0.78–0.97 | <0.001 | 0.85* | 0.65–0.94 | <0.001 | 0.62* | 0.18–0.86 | 0.007 | |
| Early Diastolic Uncoupling | 0.87* | 0.69–0.95 | <0.001 | 0.93* | 0.83–0.97 | <0.001 | 0.80* | 0.53–0.92 | <0.001 | 0.65* | 0.22–0.87 | 0.004 | |
| Late Diastolic Uncoupling | 0.76* | 0.48–0.90 | <0.001 | 0.71* | 0.37–0.88 | <0.001 | 0.85* | 0.65–0.94 | <0.001 | 0.52* | 0.05–0.81 | 0.017 | |
| Results from post-hoc sensitivity analyses showing the reliability of SVL and SAL parameters in subsets with matched cardiac cycles across apical views. ICC = Intraclass Correlation Coefficient; CI = Confidence Interval. * = *p*<.05. | | | | | | | | | | | | |  |

**Supplementary Material 4. Reliability of all left ventricular strain–volume loop parameters**

|  | ***Intra-observer (n=29)*** | | | ***Inter-observer (n=29)*** | | | ***Test–retest (n=29)*** | | | | | |
| --- | --- | --- | --- | --- | --- | --- | --- | --- | --- | --- | --- | --- |
| **Parameter** | **ICC** | **95% CI** | ***p*-value** | **ICC** | **95% CI** | ***p*-value** | **ICC** | **95% CI** | ***p*-value** | |  |  |
| Early Systolic slope | 0.68* | 0.42–0.84 | <0.001 | 0.47* | 0.14–0.71 | 0.002 | 0.34* | -0.02–0.63 | 0.032 | | |  |
| Systolic slope | 0.89* | 0.77–0.94 | <0.001 | 0.92* | 0.83–0.96 | <0.001 | 0.84* | 0.68–0.92 | <0.001 | | |  |
| Peak Strain | 0.85* | 0.71–0.93 | <0.001 | 0.64* | 0.24–0.83 | <0.001 | 0.72* | 0.49–0.86 | <0.001 | | |  |
| Early Diastolic slope | 0.80* | 0.62–0.90 | <0.001 | 0.72* | 0.48–0.86 | <0.001 | 0.44* | 0.09–0.69 | 0.008 | | |  |
| Late Diastolic slope | 0.79* | 0.60–0.90 | <0.001 | 0.56* | 0.26–0.77 | <0.001 | 0.45* | 0.11–0.70 | 0.006 | | |  |
| Uncoupling | 0.88* | 0.75–0.94 | <0.001 | 0.85* | 0.71–0.93 | <0.001 | 0.58* | 0.28–0.78 | <0.001 | | |  |
| Early Diastolic Uncoupling | 0.87* | 0.74–0.94 | <0.001 | 0.85* | 0.72–0.93 | <0.001 | 0.60* | 0.30–0.79 | <0.001 | | |  |
| Late Diastolic Uncoupling | 0.78* | 0.58–0.89 | <0.001 | 0.74* | 0.52–0.87 | <0.001 | 0.48* | 0.15–0.72 | 0.004 | | |  |
| Peak Strain / EDV | 0.82* | 0.65–0.91 | <0.001 | 0.79* | 0.61–0.90 | <0.001 | 0.74* | 0.52–0.87 | <0.001 | | |  |
| Loop area | 0.86* | 0.73–0.93 | <0.001 | 0.85* | 0.70–0.92 | <0.001 | 0.59* | 0.29–0.78 | <0.001 | | |  |
| Intra-, inter-observer and test–retest reliability of all left ventricular strain–volume loop parameters. CI = confidence interval; EDV = end-diastolic volume; ICC = intraclass correlation coefficient. * = *p*<.05. | | | | | | | | | |  |  |  |

**Supplementary Material 5. Absolute values of all left ventricular strain–volume loop parameters across analyses**

|  | ***Baseline (n=29)*** | | ***Intra-observer (n=29)*** | | ***Inter-observer (n=29)*** | | ***Test–retest (n=29)*** | |
| --- | --- | --- | --- | --- | --- | --- | --- | --- |
| **Parameter** | **Outcome** | **IQR** | **Outcome** | **IQR** | **Outcome** | **IQR** | **Outcome** | **IQR** |
| Early Systolic slope (%/mL) | 0.28 | 0.23–0.38 | 0.31 | 0.26–0.46 | 0.41* | 0.24–0.50 | 0.31 | 0.24–0.48 |
| Systolic slope (%/mL) | 0.27 | 0.23–0.33 | 0.28 | 0.24–0.32 | 0.29 | 0.22–0.34 | 0.28 | 0.23–0.35 |
| Peak Strain (%) | -18.43 | -20.72–-17.12 | -18.95 | -19.80–-17.33 | -19.76* | -21.01–-18.87 | -18.04 | -20.23–-17.49 |
| Early Diastolic slope (%/mL) | 0.37 | 0.24–0.47 | 0.37 | 0.21–0.47 | 0.34 | 0.25–0.55 | 0.45* | 0.27–0.64 |
| Late Diastolic slope (%/mL) | 0.25 | 0.20–0.36 | 0.26 | 0.18–0.37 | 0.27 | 0.19–0.38 | 0.26 | 0.21–0.33 |
| Uncoupling (%) | -0.26 | -0.78–0.34 | -0.38 | -0.85–0.43 | -0.15 | -0.68–0.17 | -0.68 | -1.03–-0.11 |
| Early Diastolic Uncoupling (%) | -0.39 | -0.92–0.26 | -0.57 | -1.05–0.37 | -0.26 | -0.90–0.12 | -0.82* | -1.29–0.16 |
| Late Diastolic Uncoupling (%) | -0.01 | -0.57–0.51 | -0.07 | -0.55–0.45 | -0.09 | -0.51–0.48 | -0.33 | -0.63–0.54 |
| Peak Strain / EDV | -0.14 | -0.18–-0.12 | -0.14* | -0.17–-0.12 | -0.16 | -0.19–-0.13 | -0.15 | -0.18–-0.11 |
| Loop area | -15.08 | -42.28–24.74 | -23.34 | -59.39–33.82 | -10.41 | -45.61–11.29 | -41.52 | -62.94–-8.94 |
| Absolute outcomes of left ventricular strain–volume loop parameters. Values represent median and interquartile range. Wilcoxon signed-rank tests evaluated differences relative to the baseline analysis (i.e., first assessment of the first TTE by observer 1). Intra-observer = repeated analysis of the first TTE by observer 1; inter-observer = independent analysis of the first TTE by observer 2; test–retest = analysis of second TTE by observer 1.  EDV = end-diastolic volume; IQR = interquartile range; TTE = transthoracic echocardiogram. * = *p*<.05. | | | | | | | | |

**Supplementary Material 6. Reliability of all right ventricular strain–area loop parameters**

|  | ***Intra-observer (n=29)*** | | | ***Inter-observer (n=29)*** | | | ***Test–retest (n=29)*** | | | | | |
| --- | --- | --- | --- | --- | --- | --- | --- | --- | --- | --- | --- | --- |
| **Parameter** | **ICC** | **95% CI** | ***p*-value** | **ICC** | **95% CI** | ***p*-value** | **ICC** | **95% CI** | ***p*-value** | |  |  |
| Early Systolic slope | 0.75* | 0.53–0.87 | <0.001 | 0.03 | -0.31–0.38 | 0.438 | 0.00 | -0.36–0.36 | 0.500 | | |  |
| Systolic slope | 0.81* | 0.63–0.90 | <0.001 | 0.89* | 0.77–0.94 | <0.001 | 0.78* | 0.58–0.89 | <0.001 | | |  |
| Peak Strain | 0.59* | 0.29–0.78 | <0.001 | 0.19 | -0.13–0.49 | 0.125 | 0.34* | -0.02–0.62 | 0.033 | | |  |
| Early Diastolic slope | 0.81* | 0.64–0.91 | <0.001 | 0.63* | 0.35–0.80 | <0.001 | 0.71* | 0.47–0.85 | <0.001 | | |  |
| Late Diastolic slope | 0.67* | 0.40–0.83 | <0.001 | 0.13 | -0.24–0.47 | 0.249 | 0.65* | 0.38–0.82 | <0.001 | | |  |
| Uncoupling | 0.70* | 0.45–0.84 | <0.001 | 0.55* | 0.24–0.76 | <0.001 | 0.60* | 0.30–0.79 | <0.001 | | |  |
| Early Diastolic Uncoupling | 0.68* | 0.42–0.84 | <0.001 | 0.54* | 0.23–0.75 | 0.001 | 0.53* | 0.20–0.75 | 0.001 | | |  |
| Late Diastolic Uncoupling | 0.64* | 0.36–0.81 | <0.001 | 0.60* | 0.31–0.79 | <0.001 | 0.73* | 0.51–0.87 | <0.001 | | |  |
| Peak Strain / EDA | 0.83* | 0.67–0.92 | <0.001 | 0.73* | 0.51–0.86 | <0.001 | 0.81* | 0.64–0.91 | <0.001 | | |  |
| Loop area | 0.59* | 0.30–0.79 | <0.001 | 0.50* | 0.18–0.73 | 0.002 | 0.53* | 0.21–0.75 | 0.001 | | |  |
| Intra-, inter-observer and test–retest reliability of all right ventricular strain–area loop parameters. CI = confidence interval; EDA = end-diastolic area; ICC = intraclass correlation coefficient. * = *p*<.05. | | | | | | | | | |  |  |  |

**Supplementary Material 7. Absolute values of all right ventricular strain–area loop parameters across analyses**

|  | ***Baseline (n=29)*** | | ***Intra-observer (n=29)*** | | ***Inter-observer (n=29)*** | | ***Test–retest (n=29)*** | |
| --- | --- | --- | --- | --- | --- | --- | --- | --- |
| **Parameter** | **Outcome** | **IQR** | **Outcome** | **IQR** | **Outcome** | **IQR** | **Outcome** | **IQR** |
| Early Systolic slope (%/cm^2^) | 1.71 | 1.34–2.37 | 1.39* | 0.84–2.28 | 2.04 | 1.56–2.79 | 1.77 | 1.04–2.02 |
| Systolic slope (%/cm^2^) | 2.42 | 1.90–2.77 | 2.21 | 1.80–2.62 | 2.42 | 2.03–2.66 | 2.18 | 1.87–2.59 |
| Peak Strain (%) | -22.54 | -23.76–-21.58 | -22.27 | -24.57–-19.61 | -24.64* | -26.03–-21.60 | -22.05 | -23.94–-20.85 |
| Early Diastolic slope (%/cm^2^) | 0.84 | -0.27–1.84 | 0.70 | -0.40–1.23 | 0.80 | 0.17–1.66 | 0.53 | -0.14–1.38 |
| Late Diastolic slope (%/cm^2^) | 2.67 | 2.04–3.89 | 2.76 | 2.12–3.77 | 2.85 | 2.30–4.06 | 2.82 | 2.45–3.44 |
| Uncoupling (%) | 2.33 | 1.54–2.96 | 2.38 | 1.74–2.94 | 1.90 | 1.52–3.19 | 2.32 | 1.18–3.26 |
| Early Diastolic Uncoupling (%) | 2.78 | 1.98–3.22 | 2.93 | 2.24–3.28 | 2.83 | 1.82–3.73 | 2.63 | 1.48–3.94 |
| Late Diastolic Uncoupling (%) | 1.29 | 0.62–2.44 | 1.36 | 0.40–2.34 | 0.90 | 0.49–1.85 | 1.35 | 0.57–1.93 |
| Peak Strain / EDA | -0.92 | -1.09–-0.76 | -0.83* | -1.01–-0.71 | -0.85 | -1.11–-0.80 | -0.90 | -1.04–-0.69 |
| Loop area | 22.51 | 15.33–30.58 | 20.18 | 18.10–28.17 | 20.10 | 14.34–31.03 | 18.94 | 12.34–32.64 |
| Absolute outcomes of right ventricular strain–area loop parameters. Values represent median and interquartile range. Wilcoxon signed-rank tests evaluated differences relative to the baseline analysis (i.e., first assessment of the first TTE by observer 1). Intra-observer = repeated analysis of the first TTE by observer 1; inter-observer = independent analysis of the first TTE by observer 2; test–retest = analysis of second TTE by observer 1.  EDA = end-diastolic area; IQR = interquartile range; TTE = transthoracic echocardiogram. * = *p*<.05. | | | | | | | | |
